# Genome-wide identification, characterization, and expression patterns analysis of the SBP-box gene family in wheat (*Triticum aestivum* L.)

**DOI:** 10.1038/s41598-020-74417-x

**Published:** 2020-10-14

**Authors:** Ying Li, Qilu Song, Yamin Zhang, Zheng Li, Jialin Guo, Xinhong Chen, Gaisheng Zhang

**Affiliations:** 1 College of Agronomy, Northwest A & F University, Yangling, 712100 Shaanxi People’s Republic of China; 2 National Yangling Agricultural Biotechnology and Breeding Center, Yangling, 712100 Shaanxi People’s Republic of China; 3 Yangling Branch of State Wheat Improvement Centre, Yangling, 712100 Shaanxi People’s Republic of China; 4 Wheat Breeding Engineering Research Center, Ministry of Education, Yangling, 712100 Shaanxi People’s Republic of China; 5 Key Laboratory of Crop Heterosis of Shaanxi Province, Yangling, 712100 Shaanxi People’s Republic of China

**Keywords:** Plant sciences, Plant development, Computational biology and bioinformatics, Data mining, Data processing, Databases, Genome informatics

## Abstract

*SQUAMOSA* promoter-binding protein (*SBP*)-box genes encode a family of plant-specific transcription factors that play roles in plant growth and development. The characteristics of SBP-box genes in rice (*Oryza sativa*) and *Arabidopsis* have been reported, but their potential roles in wheat (*Triticum aestivum*) are not fully understood. In this study, 48 *SBP*-box genes (*TaSBPs*) were identified; they were located in all wheat chromosomes except for 4B and 4D. Six *TaSBPs* were identified as tandem duplication genes that formed three tandem duplication pairs, while 22 were segmentally duplicated genes that formed 16 segmental duplication pairs. Subcellular localization prediction showed TaSBPs were located in nucleus. Among the 48 *TaSBP*s, 24 were predicted to be putative targets of *TamiR156*. Phylogenetic analysis showed that TaSBPs, AtSBPs, and OsSBPs that shared similar functions were clustered into the same subgroups. The phylogenetic relationships between the TaSBPs were supported by the identification of highly conserved motifs and gene structures. Four types of *cis*-elements––transcription-related, development-related, hormone-related, and abiotic stress-related elements––were found in the *TaSBP* promoters. Expression profiles indicated most *TaSBPs* participate in flower development and abiotic stress responses. This study establishes a foundation for further investigation of *TaSBP* genes and provides novel insights into their biological functions.

## Introduction

The *SQUAMOSA* promoter-binding protein (*SBP*)-box gene family belongs to a group of plant-specific genes that encode zinc (Zn) finger proteins. *SBP*-box genes were first discovered in *Antirrhinum majus* based on the ability of their protein products to bind to the promoter region of *SQUAMOSA*, a floral meristem identity gene^1^. Since then, most *SBP*-box genes have been identified in different plant species and their functions have been extensively investigated^2^. SBP-box transcription factors (TFs) are characterized by a highly conserved SBP domain, which contains approximately 75–79 amino acid residues and consists of two Zn finger structures along with a highly conserved nuclear localization signal region (NLS)^2^.It has been shown that the SBP domain of *SBP*-box TFs is necessary for binding to a palindromic GTAC core motif^3^.

*SBP*-box genes are known to participate in many physiological and molecular processes, including plant architecture, grain quality and yield, flowering, floral induction, and so on^4–7^. In rice, *OsSPL13* can increase grain yield by regulating the cell size in the grain hull^4^, *OsSPL14* is related to plant architecture and substantially enhances grain yield^8^, *OsSPL16* promotes grain quality and yield^9^. In *Arabidopsis, SPL3* is known to be highly expressed in vegetative and inflorescence apices, floral meristems, leaves, and floral primordials^7^; *SPL3*, *SPL4*, and *SPL5* are dramatically up-regulated in response to long-day floral induction^10^; *SPL3*/4/5 induce flowering by binding to *AP1*, *LFY*, and *FUL* promoters^11^^.^ In wheat, *TaSPL20* and *TaSPL21* govern yield-related traits in hexaploid wheat^12^ and *TaSPL8* modulates leaf angle through auxin and brassinosteroid signaling^13^, while ectopic expression of *TaSPL16* in *Arabidopsis* delays the emergence of vegetative leaves, increases organ size, and affects yield-related traits^14^. These studies have indicated that SBP-box genes function in regulation of plant development and growth.

*SBP*-box genes are also among the conserved plant TFs that are targeted by microRNAs (miRNAs), especially *miR156*/*157* family members^15^. For example, 10 *AtSBP* genes are predicted or verified to be targeted by miR156 in *Arabidopsis*^8^. In rapeseed (*Brassica napus*), 44 BnSBPs were predicted to be targeted by miR156^16^. In rice, there are 11 *SBP*-box genes that are targets of *OsmiR156*, and tissue-specific interactions have been revealed between *OsmiR156* and *OsSBP* genes^17^. However, there are few reports on whether miRNA regulation is conserved in wheat *SBP*-box genes with miRNA-binding sites.

To date, a large number of SBP-box genes have been identified in different plants: for example, there are 16 in *Arabidopsis*, 18 in rice^18^, 29 in maize^19^, and 19 in grapevines^20^. Wheat is one of the most important food crops worldwide. Compared with other plant species, the identification and functional analysis of the *SBP*-box gene family in wheat is not so advanced. In this study, we conducted genome-wide identification of *SBP*-box genes in wheat and performed a phylogenetic analysis and classified the genes into subgroups to explore the evolution of the SBP-box gene family. The exon–intron structure, the conserved motifs, and expression patterns were also analyzed. This study establishes a foundation for further analysis of *SBP*-box genes in wheat and other plants species.

## Results

### Identification of *SBP*-box genes in wheat

**Figure 1:**
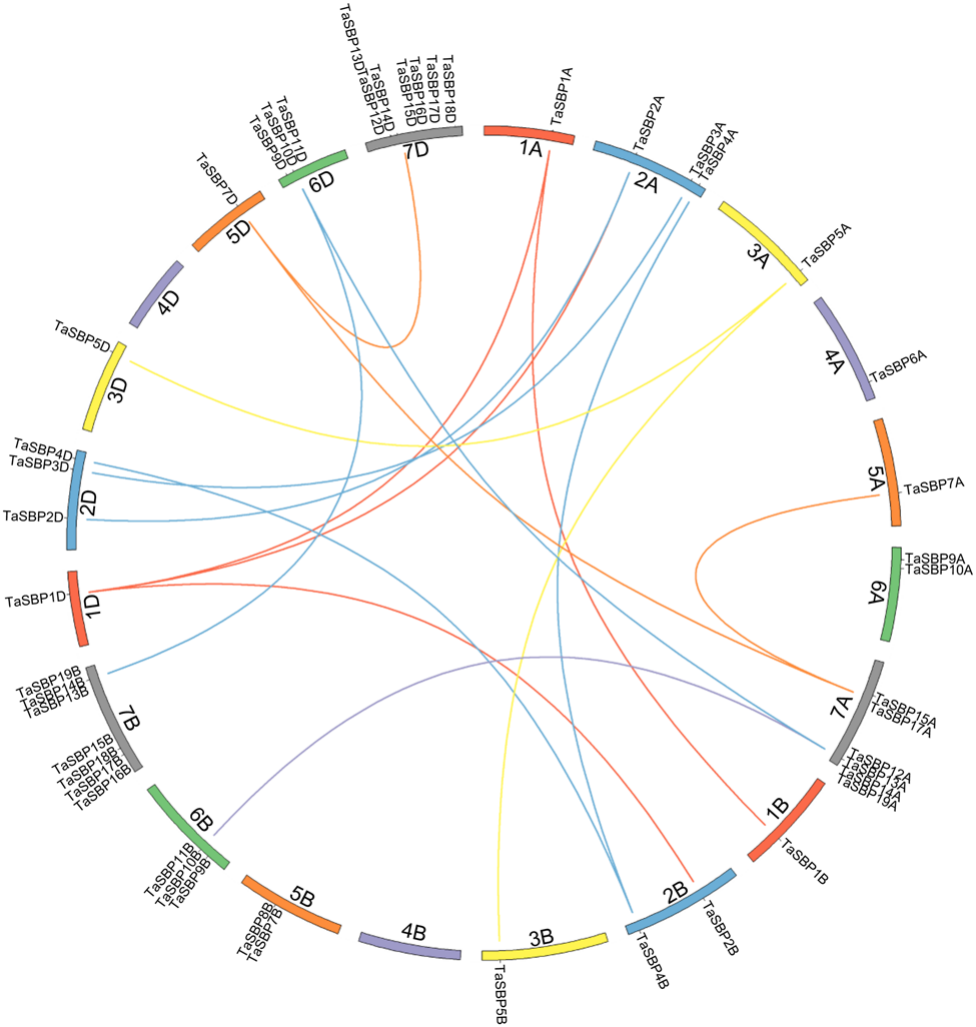
Chromosome location and duplication of *TaSBP* genes on wheat chromosomes. There are 48 TaSBPs and they are unevenly distributed in 19 wheat chromosomes except for Chr 4B and 4D. Different color lines indicated duplication pairs of *TaSBP* genes.

To identify the *SBP* genes in wheat, we performed a Hidden Markov Model (HMM) search and 48 non-redundant SBP genes were identified in the wheat genome (Supplementary Table S1). The number of hexaploid wheat SBP (*TaSBP*) genes in wheat (48) was much higher than those in rice (18), maize (31), and *Arabidopsis* (16)^18,19^. The 48 *TaSBP* genes were named *TaSBP1A* to *TaSBP19D* according to their distribution on chromosomes and genomic homology. All chromosomes contained at least one *TaSBP* gene, except for chromosomes 4B and 4D (Fig. 1). As shown in Fig. 1 and Supplementary Table S2, 22 segmentally duplicated genes were identified; these formed 16 segmental duplication pairs. Meanwhile, three tandem duplication pairs were derived from chromosomal tandem duplication. The 48 genes––with the exception of *TaSBP11A, TaSBP11B,* and *TaSBP11D*––were verified by expressed sequence tags (ESTs) deposited in the National Center for Biotechnology Information (NCBI) database, and 36 *TaSBP* genes constituted 12 sets, with every set including three homologous genes in the A, B, and D sub-genomes, respectively.

The physical features of the *TaSBP* genes were predicted. The protein length varied from 192 (TaSBP2A) to 1124 (TaSBP16D) amino acids; the isoelectric point varied from 5.73 (TaSBP6A) to 9.87 (TaSBP2D); and the molecular weight varied from 20.117 kDa (TaSBP2B) to 123.141 kDa (TaSBP16D). Detailed information is presented in Supplementary Table S1.

### Subcellular localization of TaSBP proteins

**Figure 2:**
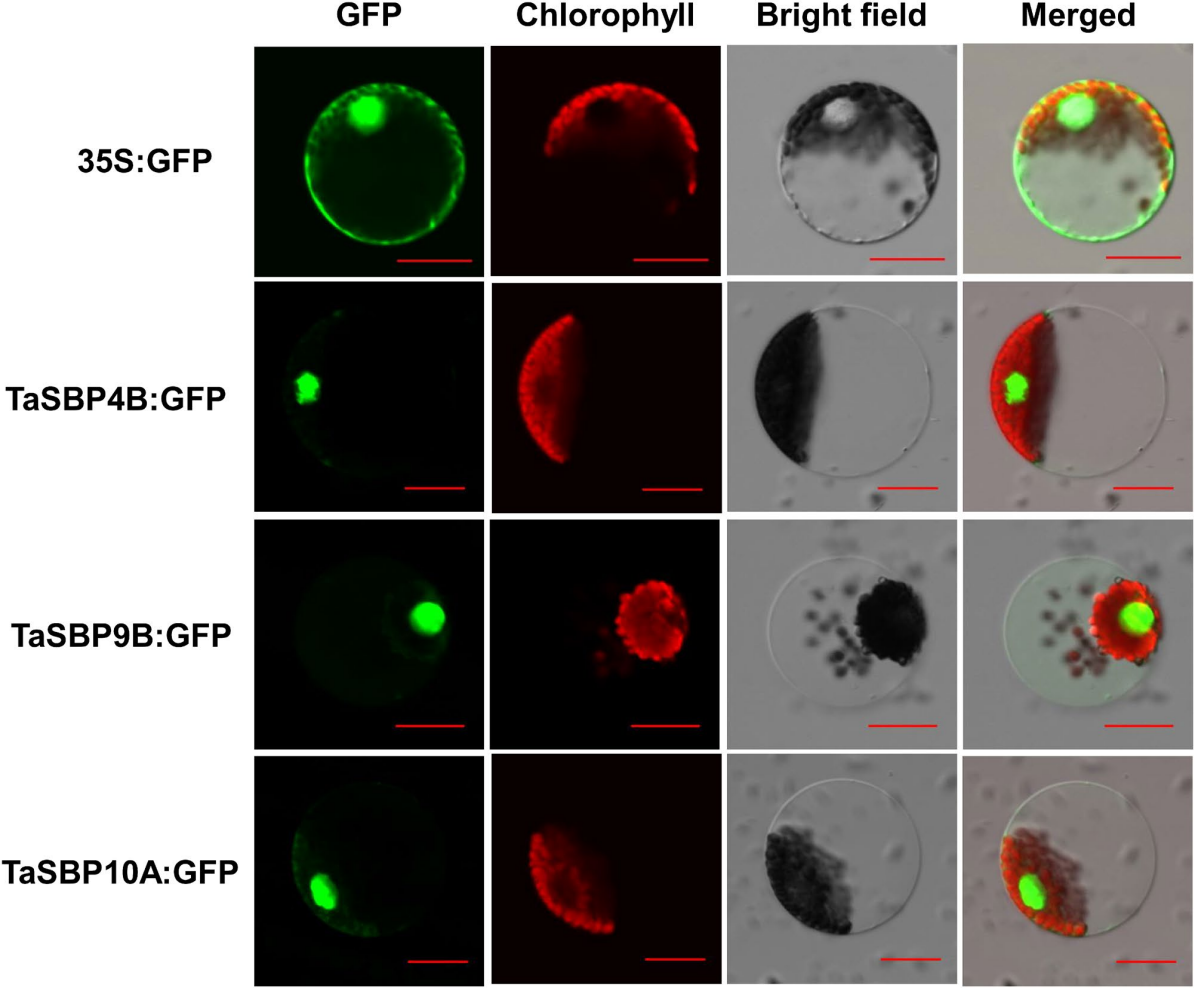
Subcellular localization of three TaSBPs. The selected TaSBP genes were cloned from Chinese Spring and used to construct CaMV35S::TaSBPs–GFP vectors in which GFP was fused at the C-terminus. The merged pictures include the green fluorescence channel and the chloroplast autofluorescence channel. Bar = 20 μm.

The results of protein subcellular localization showed that all TaSBP proteins were located in the nucleus (Supplementary Table S1). To determine if wheat TaSBPs localize to nucleus, we cloned three TaSBPs from *Chinese Spring* and assessed the subcellular localization of the encoded TaSBPs by transient expression assays in wheat protoplasts, using translational fusions to GFP. As shown in Fig. 2, all three proteins (TaSBP4B, TaSBP9B, and TaSBP10A) expressed TaSBP-GFP fusion proteins in transformed wheat protoplasts, TaSBP4B, TaSBP9B, and TaSBP10A localized on the nucleus.

### Multiple alignment and phylogenetic analysis of TaSBPs

**Figure 3:**
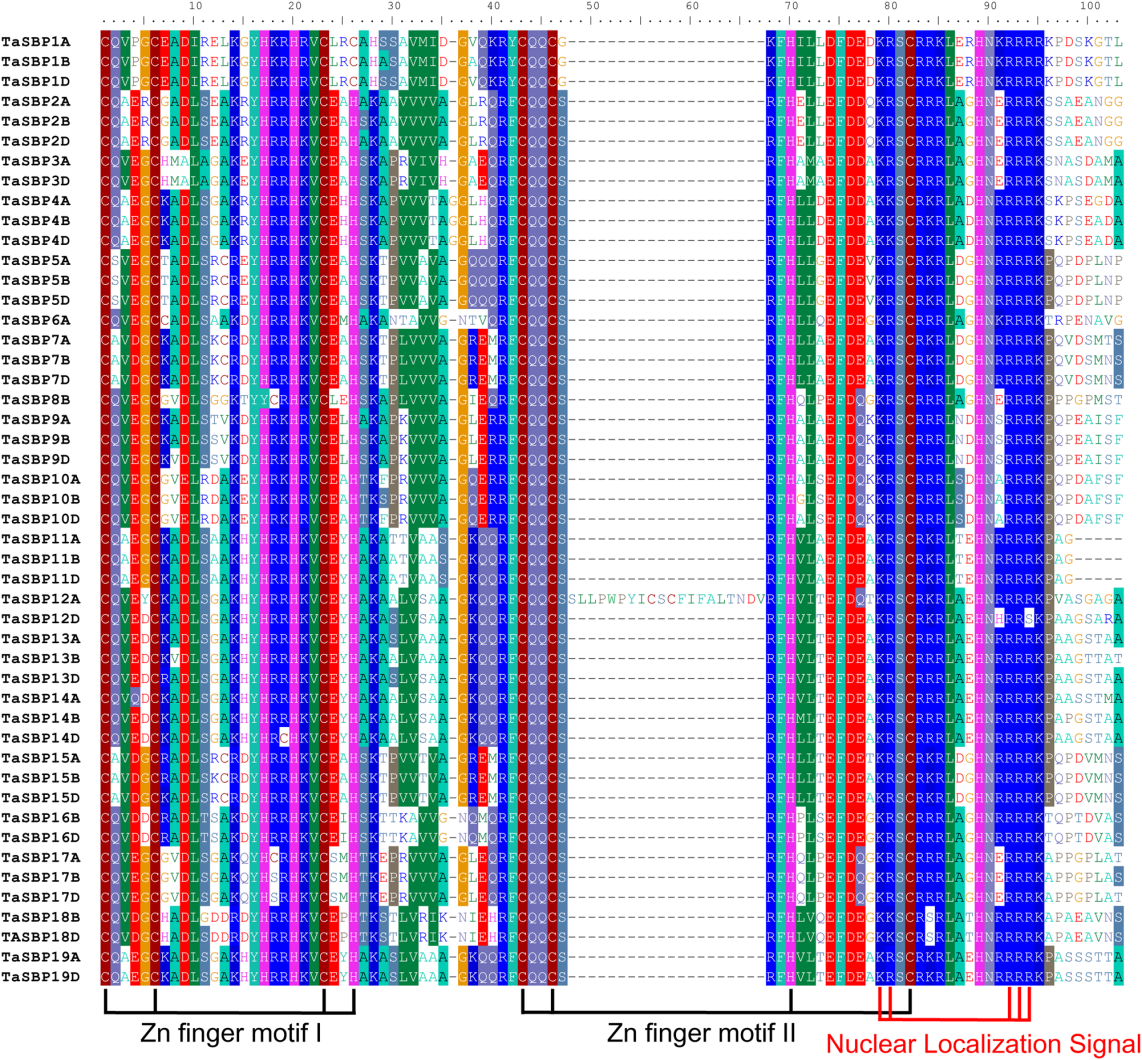
Multiple alignment of the SBP domains from the TaSBP proteins based on ClustalW program, and the two conserved zinc finger structures (Zn finger motif I and motif II) and nuclear localization signal (NLS) are demonstrated.

All the TaSBP proteins were aligned using ClustalW. As shown in Fig. 3, each SBP domain contained a complete SBP domain with two Zn finger motifs and one nuclear localization signal region (NLS). The first Zn finger motif was a CCCH type motif, and the second was a CCHC type motif.

**Figure 4:**
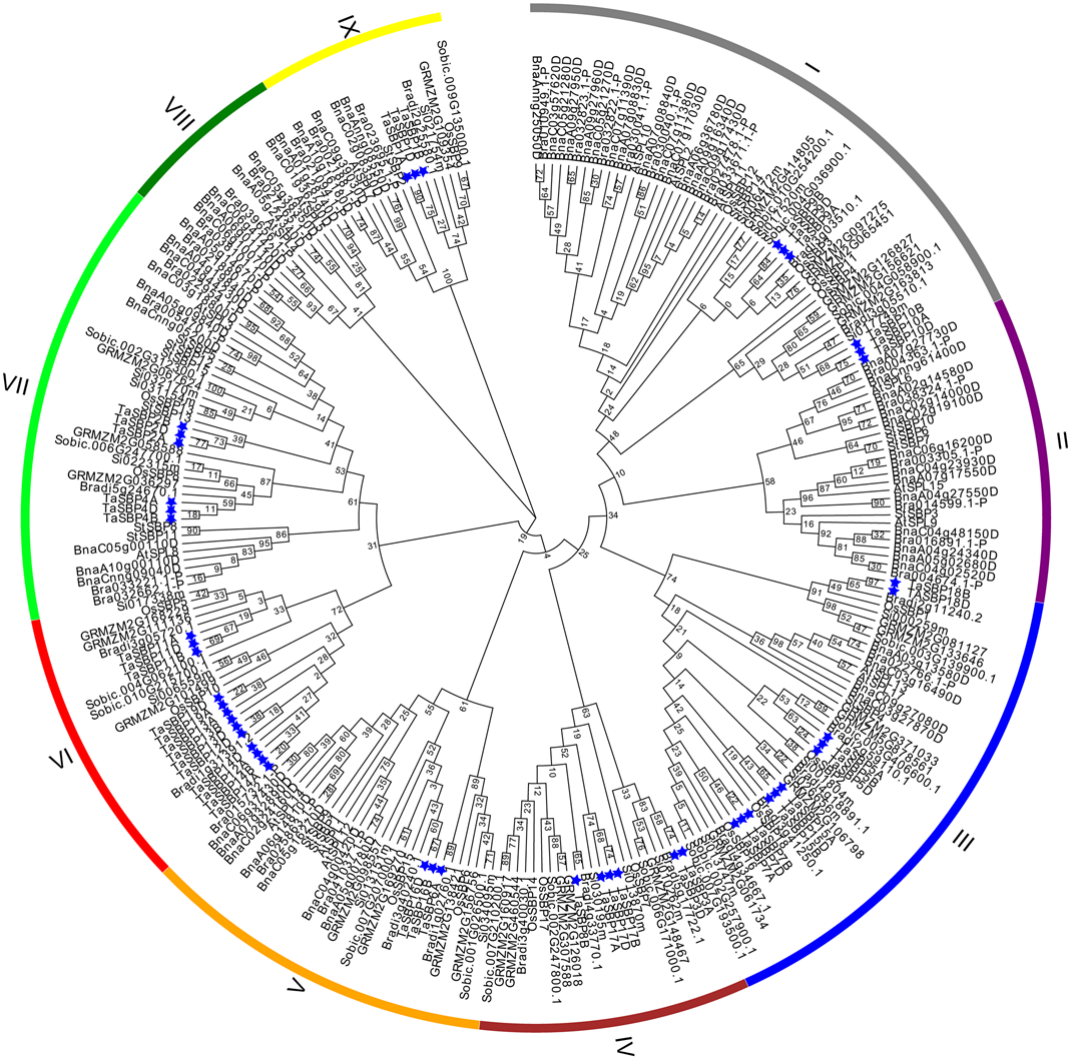
Phylogenetic relationship of SBPs. An un-rooted phylogenic tree was constructed in MEGA 7 on the basis of multiple alignment of full-sequences from six monocot species (*T. aestivum*, *O. sativa*, *Z. mays*, *S. bicolor*, *S. italica*, and *B. distachyon*) and four dicot species (*A. thaliana*, *B. napus*, *B. rapa*, and *S. tuberosum*).

**Figure 5:**
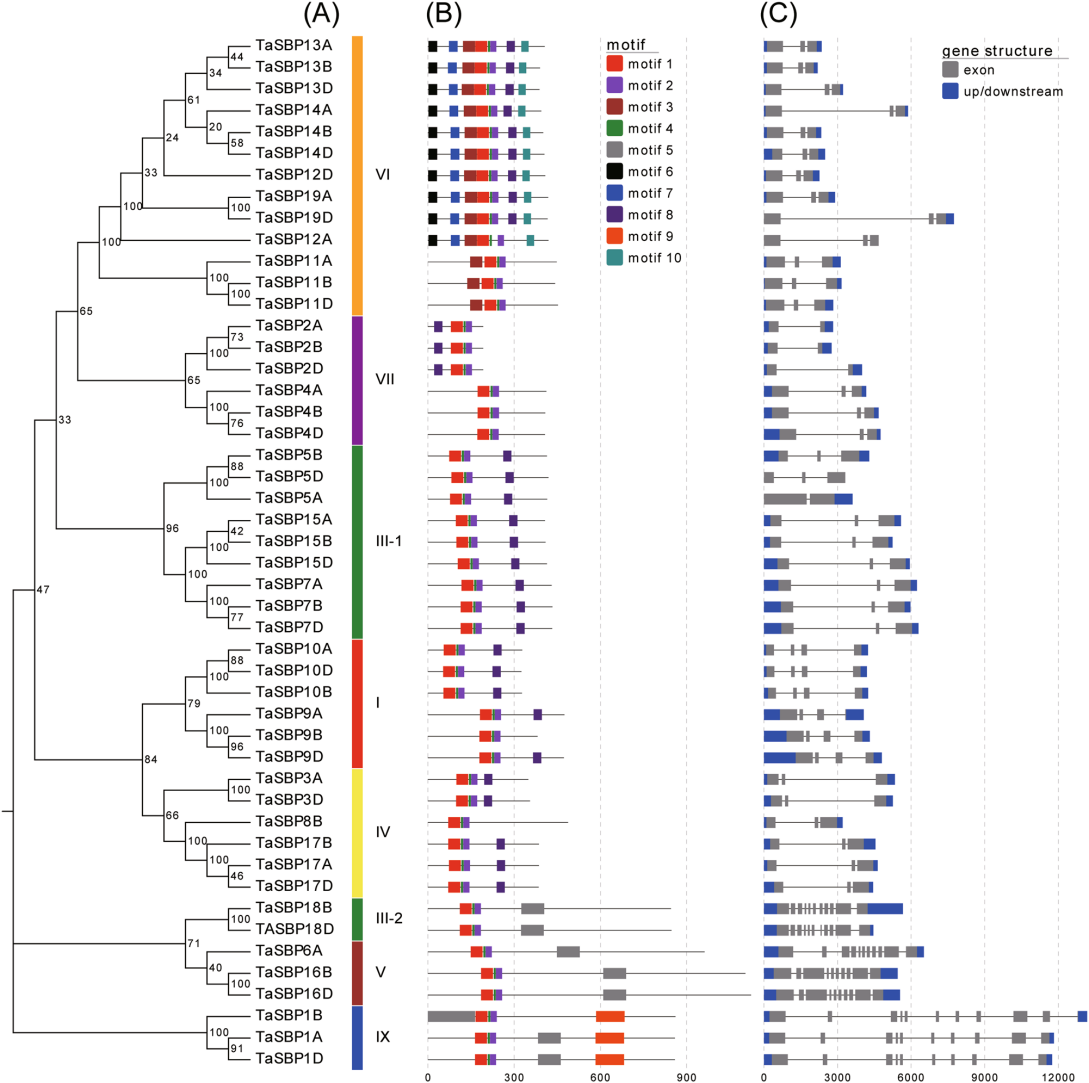
Phylogenetic relationships, conserved motifs, and gene structures of TaSBPs. (**A**) Wheat TaSBPs were classified into seven groups according to bootstrap values and the phylogenetic analysis of wheat and other plant species; (**B**) ten conserved motifs were identified in protein sequences of TaSBPs; (**C**) Gene structures. Exons and introns were indicated by boxes and lines respectively.

To further evaluate the phylogenetic relationships of TaSBPs and other plant SBPs, we selected 263 SBP sequences from ten species according to previous studies^16,18,19,21,22^, including six monocot species (48 *Triticum aestivum*, 18 *Oryza sativa*, 29 *Zea mays*, 17 *Sorghum bicolor*, 18 *Setaria italica*, and 16 *Brachypodium distachyon*) and four dicot species (16 *Arabidopsis thaliana*, 28 *Brassica rapa*, 59 *Brassica napus*, and 15 *Solanum tuberosum*), and constructed a phylogenetic tree based on the full-length protein sequences alignment. According to the phylogenetic analysis (Fig. 4), SBPs from these ten plant species could be classified into night subgroups. The largest subgroup (I) contained 48 SBP members. The smallest subgroup (VIII) contained 14 members. Except for subgroup II and VIII, the other subgroups contained at least three TaSBPs. As shown in Fig. 4, subgroup II and VIII only contained dicot SBPs, while subgroups IV and VI only had monocot SBP members. Based on the phylogenetic analysis, all TaSBP members were classified into seven subgroups (Fig. 5A).

### Conserved motifs, gene structure, and sites targeted by miR156

The SBP domain forms the core of SBP transcription factors and binds to the promoter of their downstream genes. In total, 10 conserved motifs were identified and designated motif 1 to 10 (Fig. 5B). Among them, motifs 1, 2, and 4 were in the basic region and the hinge region of the SBP domain. Motifs 3, 6, 7, and 10 were only found in subgroup V; motif 5 was only present in subgroups VI and VIII; and motif 9 was only found in subgroup VIII. The structure of the *TaSBP* genes was also examined to elucidate the gene function (Fig. 5C). The number of exons ranged from 2 to 11; subgroups I, II, III, IV, V, and VII only contained 2–4 exons, while subgroups VI and VIII had more than 10 exons. The *TaSBP* genes in the same subgroup shared similar gene structures.

**Figure 6:**
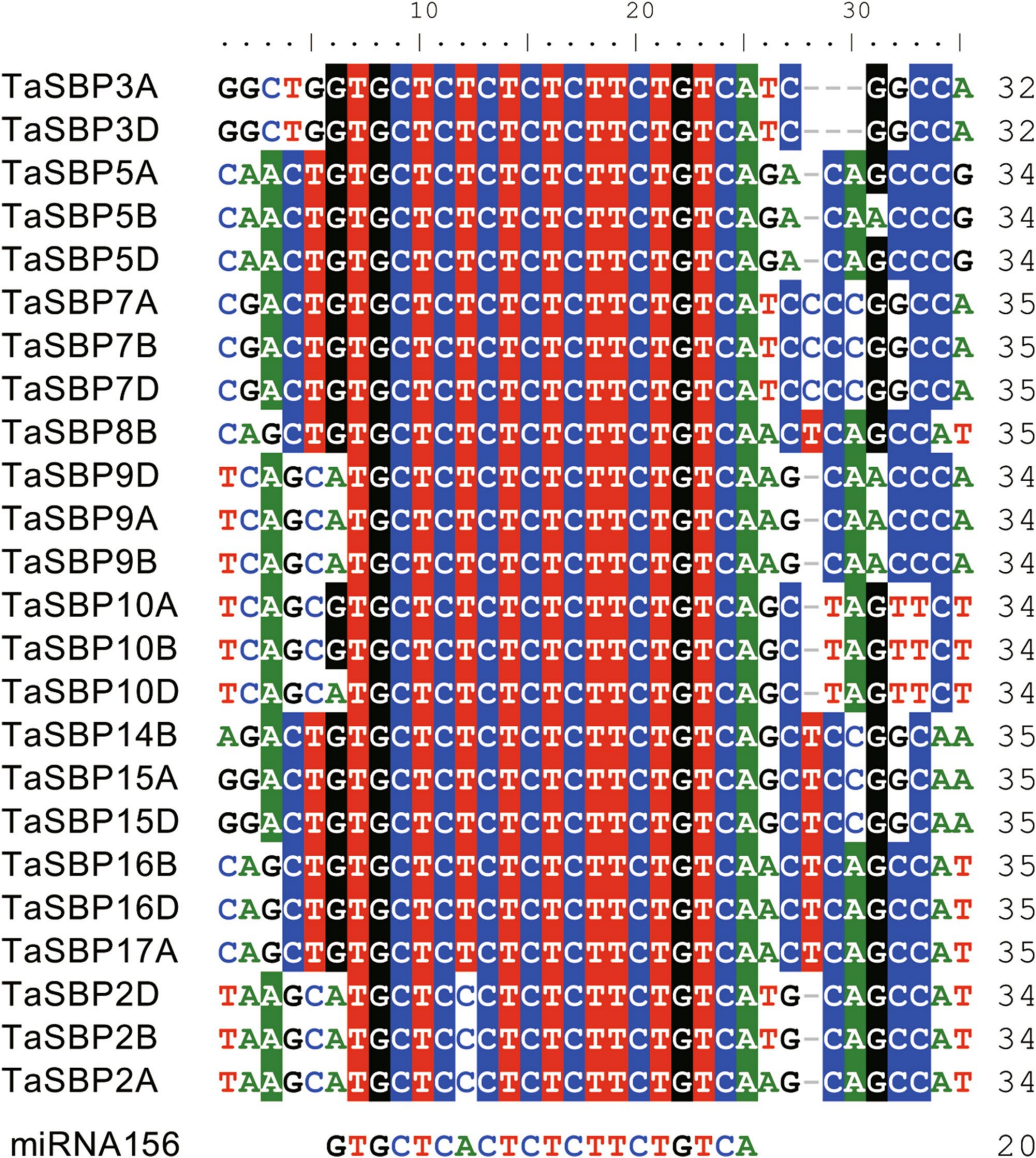
Multiple alignment of miR156 complementary sequences with the target sites in *TaSBP* genes. The target sites of 21 *TaSBPs* are located in the CDS, while 5 (*TaSBP9A, TaSBP2D, TaSBP2B, TaSBP2A, and TaSBP5A*) are located in the 3′-UTR.

To identify the miR156-mediated post-transcriptional regulation of *TaSBP* genes, we searched the coding sequences (CDSs) and 3′-untranslated region (UTR) sequences of all TaSBPs for miR156-binding sites. The results showed that 24 TaSBPs (half of the TaSBPs) had miR156-binding sites (sequences that were complementary to the mature *TamiR156* sequences), with 19 in the CDSs and 5 in the 3′-UTR regions (Fig. 6).

### *Cis*-acting elements in the promoters of *TaSBP* genes

**Figure 7:**
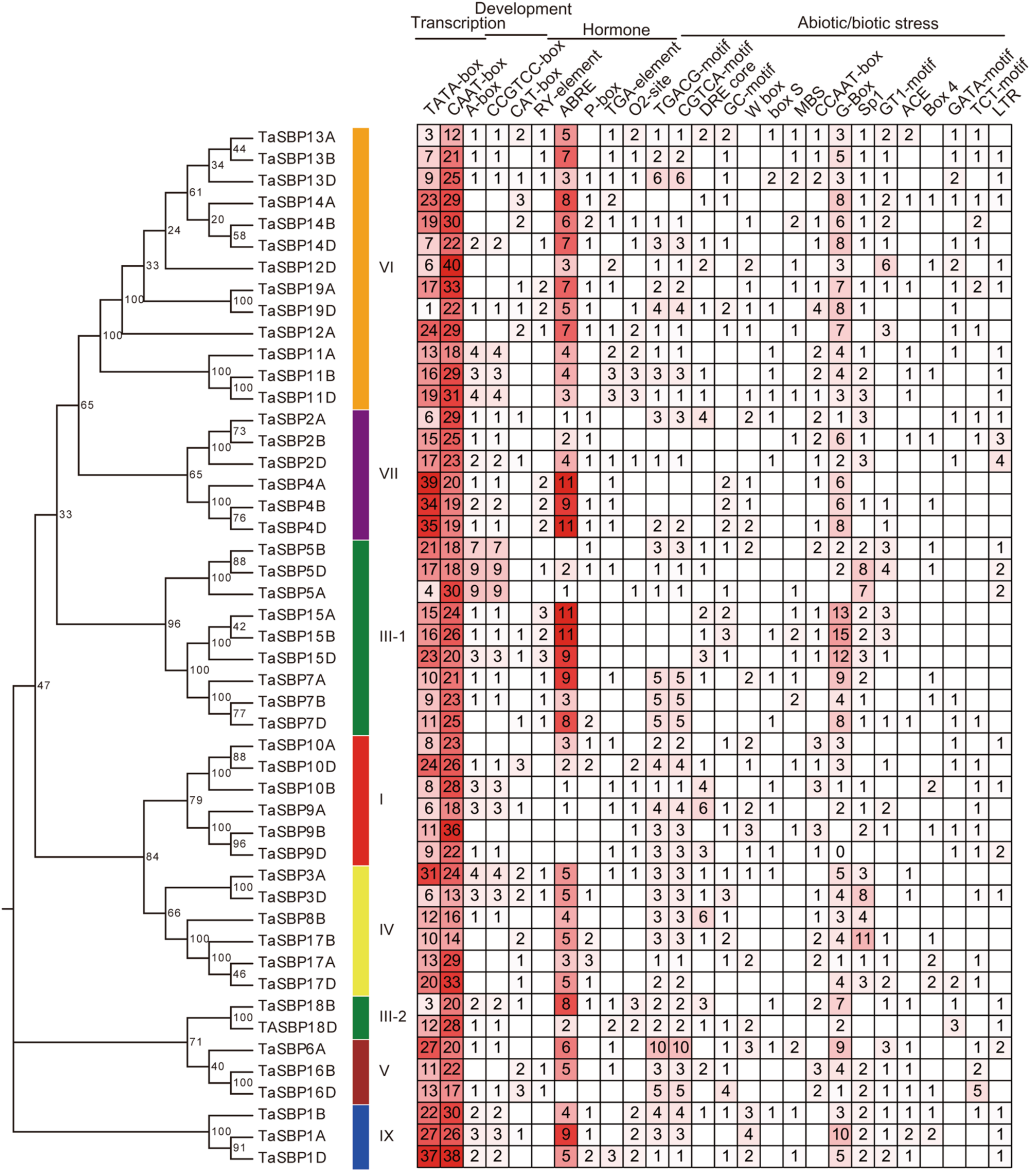
Main *cis*-elements in TaSBP gene promoters. Four types *cis*-elements––transcription-related, development-related, hormone-related, and abiotic stress-related elements––were identified in the *TaSBP* gene promoter regions. The number means the number of *cis*-elements.

*Cis*-acting elements in gene promoters are crucial regions involved in transcription factor binding for the initiation of transcription. To further explore the possible biological functions of *TaSBP* genes, the 2-kb upstream promoter regions of all *TaSBP* genes were used to predict *cis*-acting elements using the PlantCARE database. Four types of *cis*-acting elements––transcription-related, development-related, hormone-related, and abiotic stress-related elements––were identified (Fig. 7). Transcription-related *cis*-elements–including the TATA-box and CAAT-box––were found in all the *TaSBP* genes. Development-related *cis*-elements included meristem-specific regulatory elements (CCGTCC-box and CAT-box). Hormone-related *cis*-elements included the methyl jasmonate (MeJA)-responsive element (CGTC) and the abscisic acid (ABA)-responsive element (ABRE; ACGTG). Abiotic stress-related *cis*-elements included the drought response element (MYB-binding site [MBS]), low-temperature response element (LTR; CCGAAA), and anoxic specific inducibility element GC-motif (A/CGCCGCGCA). The findings indicated that the phylogenetically similar genes shared identical *cis*-elements. For example, group IV members *TaSBP4A*, *TaSBP4B*, and *TaSBP4D* had the similar proportion of *cis*-elements, group VIII members *TaSBP1A*, *TaSBP1B*, and *TaSBP1D* harbored the similar proportion of *cis*-elements.

### Analysis of the expression patterns of the *TaSBP* genes

**Figure 8:**
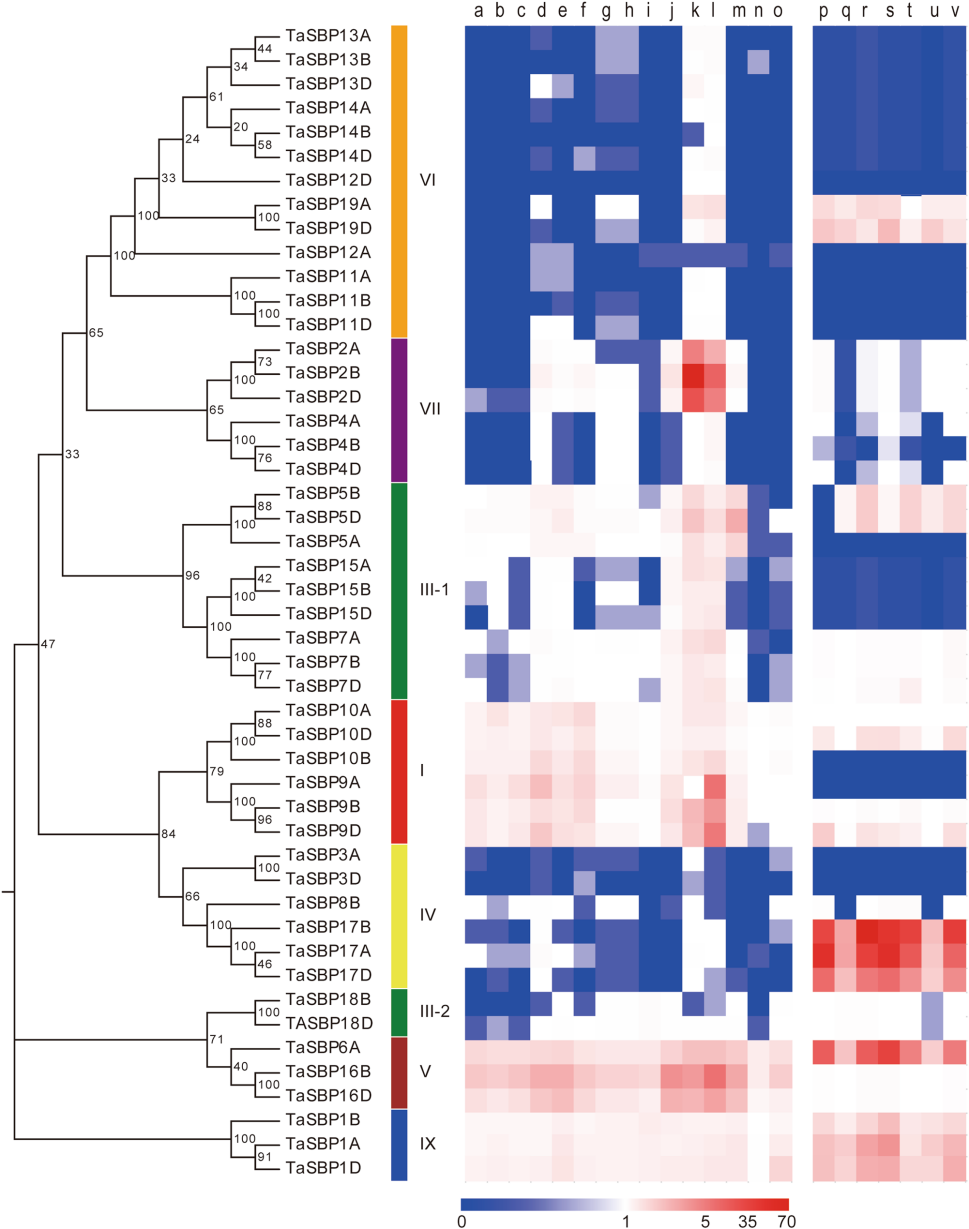
Expression profiles of TaSBP genes in different tissues and under different abiotic stresses based on transcriptome data. The 15 tissues were as follows: the root when the cotyledon emerged (**a**), three leaves were visible (**b**), and the stem reached its maximum length (**c**); the stem when two nodes or internodes were visible (**d**), half of the flowers were open (**e**), and elongation had begun (**f**); the leaf when the main shoot and axillary shoots were visible (with three nodes) (**g**), the cotyledon emerged (**h**), and the whole plant grain had formed (**i**); the inflorescence when the flowers opened (**j**), two nodes or internodes were visible (**k**), and the stem reached its maximum length (**l**); the grain when 30–50% of the whole plant grain had formed (**m**), 70–100% of the whole grain had formed (**n**), and the whole plant grain had ripened (**o**). The abiotic stresses were as follows: (**p**) normal condition, (**q**) heat stress for 1 h, (**r**) heat stress for 6 h, (**s**) drought stress for 1 h, (**t**) drought stress for 6 h, (**u**) heat and drought stress combination for 1 h, (**v**) heat and drought stress combination for 6 h.

To obtain the temporal and spatial expression patterns of *TaSBP* genes, the expression profiles were analyzed using high-throughput data from previous research^18^. As shown in Fig. 8, 95.83% (46/48) of *TaSBP* genes were detected in at least one tissue. Further, it can be seen that 89.58% were highly expressed in the inflorescence, especially when two nodes or internodes were visible and when the stem reached its maximum length.

To elucidate the roles of these *TaSBP* genes in response to abiotic stresses, expression profiles of *TaSBP* genes under different abiotic stresses were also examined. The results showed that the expression of 79.17% (38/48) of the *TaSBP* genes was detected and some of them were highly expressed in response to heat and drought stresses. The phylogenetically similar genes shared similar expression patterns. For example, the subgroup I genes had similar expression patterns, and the subgroup VIII genes were expressed in all tissues.

**Figure 9:**
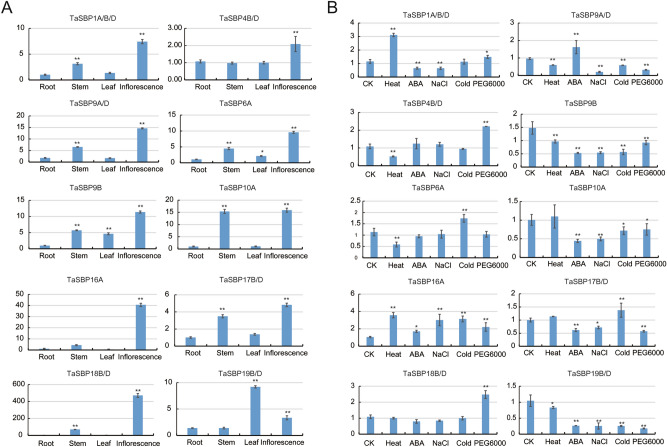
Results of quantitative RT-PCR of 10 *TaSBP* genes (**A**) in different tissues and (**B**) under different abiotic stresses. The horizontal and vertical co-ordinates represent four different tissues/abiotic stresses and the relative expression, respectively. Statistically significant differences are indicated: *P < 0.05; **P < 0.01 (Student’s t-test).

To elucidate the roles of *TaSBP* genes in wheat growth and development, we examined the relative expression levels of 10 *TaSBP* genes (each group choose at least one *TaSBP* gene) in four tissues (roots, stems, leaves, and inflorescences, collected at the heading stage) (Fig. 9A) and under different abiotic stresses (Fig. 9B). All the *TaSBP* genes were detected in at least one of the tissues examined, but different expression levels were observed. All of them were highly expressed in inflorescences. In addition, *TaSBP10A* and *TaSBP12B/D* were highly expressed in stems, and *TaSBP19B/D* was mainly expressed in leaves. These results suggested that these genes may play different roles in wheat growth and development.

To explore the potential roles of *TaSBP* genes under different abiotic stresses, seedling plants were subjected to heat, cold, ABA, salt, and drought. The changes in the transcript levels of the genes were analyzed using quantitative real-time (RT)-PCR. Results showed that all of them were induced by different abiotic stresses (Fig. 9B). The expression level of *TaSBP9B* and *TaSBP19B/D* were significantly down-regulated under different abiotic stresses when compared to control. Under heat treatment, the expression levels of TaSBP1A/B/D and 16A were significantly up-regulated. Under cold stress, the expression levels of TaSBP6A, TaSBP16A, and TaSBP17B/D was significantly up-regulated, while *TaSBP9A/D*, *TaSBP9B*, *TaSBP10A*, and *TaSBP19B/D* were significantly down-regulated*.*

## Discussion

### Characteristics of *SBP*-box genes in wheat

The SBP-box proteins are characterized by a conserved SBP domain containing about 75 amino acids and they constitute a large family of transcription factors in plants. It has been proposed that SBP-box genes are plant specific^3^. In the present study, 48 wheat SBP-box genes were identified, accounting for 0.63% of all wheat annotation genes, which is more than that in rice (0.45%), *Arabidopsis* (0.58%), maize (0.57%), and *B. distachyon* (0.51%)^18,19,23^. In terms of sub-genomes, there are 16, 16, and 16 members in wheat A, B, and D sub-genomes respectively, this number in each sub-genome is similar with the result in rice (18), *S. bicolor* (17), *S. italica* (18), and *B. distachyon* (16)^18,23,24^. Duplication analysis showed that there were 16 segmental duplication pairs were formed by 22 TaSBPs, and three tandem duplication pairs were constructed by 6 TaSBPs. Duplication promoted TaSBPs gene expansion, maybe is the reason why the number of TaSBPs is more than other plant species.

Multiple sequence analysis showed that each of TaSBPs contained two Zn finger motifs, CCCH and CCHC type motifs, and one NLS region in the SBP domain, they constitute the main identifying characteristics of SBP-box proteins. Genes within the same phylogenetic subgroup shared a similar length, gene structure, and motif composition. For example, all subgroup I, II, IV, and VII members had three exons, motifs 3, 6, 7, and 10 were only found in subgroup V. Therefore, the similar gene structure and motif composition of SBP-box genes in wheat might reflect their evolutionary relationships. In the promoters of these *TaSBP*-box genes, four kinds of *cis*-acting elements––transcription-related, development-related, hormone-related, and abiotic stress-related elements––were detected, and phylogenetically similar genes shared the same *cis*-elements.

In addition, phylogenetic analysis revealed that the TaSBP-box proteins had a close evolutionary relationship with other plant SBP-box genes, especially with monocot plants. SBPs from ten plant species could be classified into night subgroups (Fig. 4), and there are two subgroups, II and VIII, only contained dicot plant SBPs, while IV and VI only had monocot SBP members, indicated that the closer relationship between monocot plant SBPs.

### *SBP*-box genes play important roles in plant development and growth

*SBP*-box genes control a large range of processes underlying flower growth and development. For example, in *Arabidopsis,* constitutive expression of *AtSPL3* results in very early flowering and frequent changes in morphology^25^; *AtSPL2*, *AtSPL10*, and *AtSPL11* in *Arabidopsis* control morphological changes associated with shoot maturation in the reproductive phase^26^; *AtSPL8* affects pollen sac development and also controls gynoecium development^27^; the *miR156*-*SPL3* module controls *FLOWERING LOCUST* expression to regulate ambient temperature-responsive flowering^28^. In rice, *OsSPL14* is highly expressed in the reproductive stage and promotes flower development; it also affects panicle branching^29^; *OsSPL8* (*OsLG1*) controls ligule development and inflorescence architecture^30,31^. In the tomato plant, *LeSPL*/*CNR* is crucial for normal fruit development and ripening^32^. In wheat, *TaSPL20* and *TaSPL21*, corresponding to *TaSBP13D* and *TaSBP11D* in this study, respectively, were highly expressed in the lemma and palea^12^. Ectopic expression of *TaSPL20* or *TaSPL21* in rice revealed that these genes have similar functions in regard to increasing the number of primary branches, secondary branches, grain number, and panicle length^12^. Ectopic expression of *TaSPL16* (*TaSBP15B* in this study) in *Arabidopsis* delays the emergence of vegetative leaves, promotes early flowering, increases organ size, and affects yield-related traits^14^. *TaSPL8* (*TaSBP4D* in this study) in wheat affects lamina joint development and plant architecture^13^. In this study, we found that 89.58% of *TaSBP* genes were highly expressed in the inflorescence according to ArrayExpress data, especially when two nodes or internodes were visible and when the stem reached its maximum length. Additionally, in our quantitative RT-PCR analysis of different tissues, all of the 10 selected *TaSBP* genes were highly expressed in flowers. These results suggest that *TaSBP* genes might play important roles in plant development and growth.

### Conservation of miR156-binding sites in *SBP*-box genes

miRNAs play key roles in regulating the transcription of target genes. Most studies show that overexpression of miR164, miR159a, miR319, miR319, and miR399 affect members of the NAC, MYB, TCP, GAMYB, and WRKY transcription factor families, respectively^13^. Regarding the SBP-box gene family, tissue-specific interactions between OsmiR156 and *OsSBP* genes were found in rice^17^. Previous studies indicated that the miR156 function important in plant development and growth. For example, miR156 directly repressed the expression of SBP-box genes that function in juvenile-to-adult transition in wheat and *Arabidopsis*^33,34^. miR156 also play important roles in controlling flowering, leaf development, plant architecture by targeting SBP-box genes. For example, overexpression of *miR156* delays *Arabidopsis* flowering and decreases apical dominance by regulating SBP-box genes^35^. In wheat, overexpression of tae-miR156 leads to increased tiller number and severe defects in spikelet formation^36^.

In the present study, target prediction showed that 24 TaSBPs have miR156-binding sites and that phylogenetically similar genes shared the same miR156-binding site. SBP-box genes with a miRNA-binding site existed across many subgroups (I, III, IV, VI, and VII) in wheat, suggesting conservation of miRNA-binding sites because of their functional importance. Previous study showed that tae-miR156-TaSPL3/17 interact with *DWARF53* to regulate *TEOSINTE BRANCHED1 *(*TaTB1*) and *BARREN STALK1 *(*TaBA1*) expression, thus regulated wheat tillering and spikelet development^36^. Wheat *TaSPL16* (*TaSBP15B* in this study) gene have miR156-binding sites in their terminal exons^14^. It has been reported that *miR156* is responsible for the temporal expression of *SPL13* during vegetative development^35^. As in *Arabidopsis* and *Brassica napus*, a previous study reported that the homologous genes in wheat are predicted to be targets of miR156^16^. Moreover, in the present study, the sites complementary to miR156 were located in the CDS of 19 *TaSBP* genes and in the 3′UTR regions of five *TaSBP* genes. These results showed that the miR156-binding site in *SBP*-box genes is conserved across plant species.

## Materials and methods

### Data retrieval and identification of *SBP*-box genes

To identify the *SBP*-box genes in wheat, the HMMER profile of the SBP-box-binding domain (PF03110) was obtained from the Pfam database (https://pfam.xfam.org/) and searched against the protein sequences of wheat with a threshold of e < 1e^−5^. The SBP-box protein sequences of 16 *Arabidopsis* and 19 rice *SBP*-box genes^18^ were retrieved from the Ensembl Plants database, and used to conduct a BLASTP search against the protein sequences of wheat with the threshold of e < 1e^−5^ and identity of 50%. After BLASTP, a self-blast and manual correction was performed to remove the alternative splicing events and redundancy. Finally, the NCBI-Conserved Domains Database (CDD; https://www.ncbi.nlm.nih.gov/cdd) and Simple Modular Architecture Research Tool (SMART) program (https://smart.embl.de/) were used to confirm the putative SBP-box proteins. The subcellular location of *SBP*-box genes was predicted using the CELLO web tool (https://cello.life.nctu.edu.tw/). The theoretical isoelectric point and molecular weight of SBP-box genes were predicted using the ExPASy tool (https://www.expasy.org/). To further verify the existence of *TaSBP* genes in wheat, we performed BLASTN^37^ to search for expressed sequence tags (ESTs) using the CDSs of *TaSBP* genes.

The protein sequences, cDNA sequences, DNA sequences, upstream 2-kb genomic DNA sequences, and CDSs of *SBP*-box genes used in this study were downloaded from the Ensembl Plants database (https://plants.ensembl.org/index.html) for further analysis.

### Subcellular localization of *TaSBPs*

To check the subcellular localization of TaSBP4B, TaSBP9B, and TaSBP10A protein in wheat protoplast, a number of GFP fusion proteins were constructed. The cDNAs of full-length *TaSBP4B*,* TaSBP9B*, and* TaSBP10A* were cloned in frame with GFP to generate the constructs, respectively. These recombinant plasmids were transformed into wheat protoplasts using a polyethylene glycol (PEG)-mediated transient transformation system^38^. Visualization of the fluorescent proteins was performed using an Olympus IX83-FV1200 confocol microscope with excitation wavelengths of 460/480 nm for GFP and 633 nm for chloroplast.

### Chromosomal location of *TaSBP* genes and gene duplication

The chromosome distribution information of *TaSBP* genes was obtained from the Ensembl Plants database (https://plants.ensembl.org/index.html). Duplications of *TaSBP* genes were analyzed using MCScanx^39^ with E-value < 1 × 10^−10^. Tandem duplication events were defined as adjacent homologous *TaSBP* genes on the same chromosome with no more than one intervening gene, while the segmental duplication events were generated through polyploidy and chromosome rearrangements^40^. The chromosome locations and gene duplications were visualized using Circos^41^.

### Multiple sequence alignment and phylogenetic tree construction

The full-length amino acid sequences of the TaSBPs were used for multiple sequence alignment and phylogenetic analysis. Multiple sequence alignments of amino acid sequences were performed using the ClustalW program^42^ with default parameters. An un-rooted neighbor-joining tree was constructed with 1000 bootstrap replications using MEGA 7.0 software based on the full-length protein sequence alignment^43^.

### Gene structure and conserved motif analyses

The exon–intron structure of *TaSBP* genes was graphically displayed using the Gene Structure Display Server^44^ using the CDSs and DNA sequences of *TaSBPs*. The amino acid sequences of TaSBPs were used to predict the conserved motifs using the MEME Suite web server^45^ with the maximum number of motifs set at 10 and the optimum width of motifs from 5 to 200 amino acids.

### *Cis*-element analyses and miR156-binding site prediction

The upstream 2-kb genomic DNA sequences of *TaSBP* genes were submitted to the PlantCARE database (https://bioinformatics.psb.ugent.be/webtools/plantcare/html/) to identify the *cis*-elements in the *TaSBP* gene promoters. To predict the putative *miR156-*binding sites, the full lengths of *TaSBP* genes including CDS and UTR sequences were analyzed using the psRNATarget tool (https://plantgrn.noble.org/psRNATarget/?function).

### Expression patterns of *TaSBP* genes

High-throughput sequencing data for wheat were obtained from the ArrayExpress database (https://www.ebi.ac.uk/arrayexpress) under accession number E-MTAB-4484^46^. These data were used to analyze the expression profiles of *TaSBP* genes in 15 tissues, i.e. the root when the cotyledon emerged, three leaves were visible, and the stem reached its maximum length; the stem when the nodes or internodes were visible, half of the flowers were open, and elongation had begun; the leaf when the main shoot and axillary shoots were visible (with three nodes), the cotyledon emerged, and the whole plant fruit had formed; the inflorescence when the flowers opened, two nodes or internodes were visible, and the stem reached maximum length; and the grain when 30–50% of the whole plant grain had formed, 70–100% of the whole plant grain had formed, and the whole plant grain had ripened.

### Plant growth and stress treatment

The wheat cultivar Chinese Spring was planted in an artificial climate chamber at 24/22 °C (day/night) with a photoperiod of 16/8 h (day/night). Roots, stems, leaves, and inflorescences were collected at the heading stage and used for tissue analyses. For abiotic stress, 10-day-old seedlings were subjected to heat (42 °C), cold (4 °C), drought (20% polyethylene glycol [PEG] 6000), salt (200 mM NaCl), and ABA (100 μmol ABA). Subsequently, whole seedlings (including root and leaves) were collected for RNA isolation.

### RNA extraction, cDNA synthesis, and quantitative RT-PCR

Total RNA was extracted using a TIANGEN RNA Extraction Kit (Beijing, China) according to the manufacturer’s protocol. First-strand cDNA was synthesized using FastKing gDNA Dispelling RT SuperMix (Beijing, China). To assess gene expression, quantitative RT-PCR was performed on a Thermal Cycler Dice Real Time System III (ThermoFisher Scientific, China). Three biological replications were performed for each sample and 15-μL reaction systems containing 7.5 μL SYBR Premix Ex Taq (TAKARA), 0.75 μL (10 pmol/μL) each of forward and reverse primers, 0.5 μL of cDNA (200 ng/μL), and 5.5 μL of H_2_O. The quantitative RT-PCR was performed according to our previous study^47^. To normalize the total amount of cDNA present in each reaction, the wheat *ACTIN* gene was co-amplified as an endogenous control for calibration of the relative expression^48^. The relative expression level was calculated using the 2^−△△CT^ method^49^. The primers for ten sets of *TaSBP* genes (*TaSBP1A/B/D*, *TaSBP4B*/*4D*, *TaSBP6A*, *TaSBP9A*/*9D*, *TaSBP9B*, *TaSBP10A*, *TaSBP16A*, *TaSBP17B*/*17D*, *TaSBP18B*/D, and *TaSBP19B*/*19D*) were universal in each set, because of the highly conserved sequences in the A, B, and D sub-genomes. Thus, the detected expression represented a combination of up to three homologous genes. The primers are listed in Supplementary Table S3.

## Conclusions

In this study, we systematically identified *TaSBP* genes in the wheat genome. Forty-eight TaSBPs were identified and each contained a conserved SBP-box domain. The chromosome locations, gene and protein structures, subcellular localization, phylogenetic relationships, miR156-binding sites, and *cis*-elements were also characterized. The *TaSBP* expression levels in different tissues indicated that they were responsible for flower development. Quantitative RT-PCR analysis showed that the tested *TaSBP* genes were highly expressed in inflorescences and in response to abiotic stressors. This study establishes a foundation for further investigation of *TaSBP* genes and provides novel insights into their biological functions.

## Supplementary information

Supplementary Information.

## References

[1] Klein J, Saedler HP (1996). A new family of DNA binding proteins includes putative transcriptional regulators of the *Antirrhinum majus* floral meristem identity gene SQUAMOSA. Mol. Gen. Genet..

[2] Birkenbihl R, Jach GH, Huijser P (2005). Functional dissection of the plant-specific SBP-domain: Overlap of the DNA-binding and nuclear localization domains. J. Mol. Biol..

[3] Cardon G, Höhmann S, Klein J, Nettesheim K, Saedler H, Huijser P (1999). Molecular characterisation of the Arabidopsis SBP-box genes. Gene.

[4] Si LZ, Chen JY, Huang XH, Gong H, Luo JH, Hou QQ, Zhou TY, Lu TT, Zhu JJ, Shangguan YY (2016). OsSPL13 controls grain size in cultivated rice. Nat. Genet..

[5] Jiao YQ, Wang YH, Xue DW, Wang J, Yan MX, Liu GF, Dong GJ, Zeng DL, Lu ZF, Zhu XD, Qian Q, Li JY (2010). Regulation of OsSPL14 by OsmiR156 defines ideal plant architecture in rice. Nat. Genet..

[6] Luo L, Li WQ, Miura K, Ashikari M, Kyozuka J (2012). Control of tiller growth of rice by OsSPL14 and Strigolactones, which work in two independent pathways. Plant Cell Physiol..

[7] Cardon GH, Höhmann S, Nettesheim K, Saedler H, Huijser P (2012). Functional analysis of the *Arabidopsis thaliana* SBP-box gene SPL3: A novel gene involved in the floral transition. Plant J..

[8] Kazuhiko Y, Takanori K, Makoto I, Masaru T, Tomoko Y, Takashi Y, Masaaki A, Eiko S, Takayoshi M, Emi N (2004). A novel zinc-binding motif revealed by solution structures of DNA-binding domains of Arabidopsis SBP-family transcription factors. J. Mol. Biol..

[9] Wang SK, Wu K, Yuan QB, Liu XY, Liu ZB, Lin XY, Zeng RZ, Zhu HT, Dong GJ, Qian Q, Zhang GQ, Fu XD (2012). Control of grain size, shape and quality by *OsSPL16* in rice. Nat. Genet..

[10] Jung JH, Lee HJ, Ryu JY, Park CM (2016). SPL3/4/5 Integrate developmental aging and photoperiodic signals into the FT-FD module in Arabidopsis flowering. Mol. Plant..

[11] Wang JW, Czech B, Weigel D (2009). miR156-Regulated SPL transcription factors define an endogenous flowering pathway in *Arabidopsis thaliana*. Cell.

[12] Zhang B, Xu WN, Liu X, Mao XG, Li A, Wang JY, Chang XP, Zhang XY, Jing RL (2017). Functional conservation and divergence among homoeologs of *TaSPL20* and *TaSPL21*, two *SBP*-box genes governing yield-related traits in hexaploid wheat. Plant Physiol..

[13] Liu KY, Cao J, Yu KH, Liu XY, Gao YJ, Chen Q, Zhang WJ, Peng HR, Du JK, Xin MM, Hu ZR, Guo WL, Rossi V, Ni ZF, Sun QX, Yao YY (2019). Wheat *TaSPL8* modulates leaf angle through auxin and brassinosteroid signaling. Plant Physiol..

[14] Cao RF, Guo LJ, Ma M, Zhang WJ, Liu XL, Zhao HX (2019). Identification and functional characterization of squamosa promoter binding protein-like gene TaSPL16 in wheat (*Triticum aestivum* L.). Front. Plant Sci..

[15] Lakhotia N, Joshi G, Bhardwaj AR, Katiyar-Agarwal S, Agarwal M, Jagannath A, Goel S, Kumar A (2014). Identification and characterization of miRNAome in root, stem, leaf and tuber developmental stages of potato (*Solanum tuberosum* L.) by high-throughput sequencing. BMC Plant Biol..

[16] Cheng HT, Hao MY, Wang WX, Mei DS, Tong CB, Wang H, Liu J, Fu L, Hu Q (2016). Genomic identification, characterization and differential expression analysis of SBP-box gene family in *Brassica napus*. BMC Plant Biol..

[17] Xie KB, Wu CQ, Xiong LZ (2006). Genomic organization, differential expression, and interaction of *SQUAMOSA* promoter-binding-like transcription factors and microRNA156 in rice. Plant Physiol..

[18] Yang ZF, Wang XF, Gu SL, Hu ZQ, Hua X, Xu CW (2008). Comparative study of *SBP*-box gene family in *Arabidopsis* and rice. Gene.

[19] Zhang W, Bei LI, Bin YU (2016). Genome-wide identification, phylogeny and expression analysis of the *SBP*-box gene family in maize (*Zea mays*). J. Integr. Agric..

[20] Wang Y, Hu Z, Yang Y, Chen X, Chen G (2010). Genome-wide identification, phylogeny, and expression analysis of the *SBP*-box gene family in grapevine. Russ. J. Plant Physiol..

[21] Kavas M, Kızıldoğan AK, Abanoz B (2017). Comparative genome-wide phylogenetic and expression analysis of SBP genes from potato (*Solanum tuberosum*). Comput. Biol. Chem..

[22] Song S, Zhou HY, Sheng SB, Cao M, Li YY, Pang XM (2017). Genome-wide organization and expression profiling of the SBP-box gene family in Chinese Jujube (*Ziziphus jujuba* Mill.). Int. J. Mol. Sci..

[23] Davidson RM, Malali G, Gaurav M, Haining L, Brieanne V, Shin-Han S, Ning J, Buell C, Robin (2012). Comparative transcriptomics of three Poaceae species reveals patterns of gene expression evolution. Plant J..

[24] Chang JZ, Yan FX, Qiao LY, Zheng J, Zhang FY, Liu QS (2016). Genome-wide identification and expression analysis of SBP-box gene family in *Sorghum bicolor* L.. Hereditas..

[25] Gandikota M, Birkenbihl RP, Höhmann S, Cardon GH, Saedler H, Huijser P (2010). The miRNA156/157 recognition element in the 3′ UTR of the *Arabidopsis SBP* box gene *SPL3* prevents early flowering by translational inhibition in seedlings. Plant J..

[26] Masahito S, Tomotsugu K, Nobutaka M, Masaru OT (2009). *Arabidopsis SBP*-box genes *SPL10*, *SPL11* and *SPL2* control morphological change in association with shoot maturation in the reproductive phase. Plant Cell Physiol..

[27] Unte US, Anna-Marie S, Paolo P, Madhuri G, Dario L, Heinz S, Peter H (2003). *SPL8*, an *SBP*-box gene that affects pollen sac development in *Arabidopsis*. Plant Cell..

[28] Kim JJ, Lee JH, Kim WH, Jung HS, Huijser P, Hoon, Ahn JH (2012). The microRNA156-*SQUAMOSA PROMOTER BINDING PROTEIN-LIKE3* module regulates ambient temperature-responsive flowering via *FLOWERING LOCUS T* in *Arabidopsis*. Plant Physiol..

[29] Miura K, Ikeda MA, Song XJ, Ito M, Asano K, Matsuoka M, Kitano H, Ashikari M (2010). *OsSPL14* promotes panicle branching and higher grain productivity in rice. Nat. Genet..

[30] Lee J, Park JJ, Song LK, Yim J, An G (2007). Mutations in the rice liguleless gene result in a complete loss of the auricle, ligule, and laminar joint. Plant Mol. Biol..

[31] Ishii T, Numaguchi K, Miura K, Yoshida Y, Thanh PT, Htun HM, Yamasaki M, Komeda N, Matsumoto T, Terauchi R (2013). *OsLG1* regulates a closed panicle trait in domesticated rice. Nat. Genet..

[32] Kenneth M, Mahmut TR, Mervin P, Yiguo H, Thompson AJ, King GJ, Giovannoni JJ, Seymour GB (2006). A naturally occurring epigenetic mutation in a gene encoding an SBP-box transcription factor inhibits tomato fruit ripening. Nat. Genet..

[33] Liu HH, Tian X, Li YJ, Wu CA, Zheng CC (2008). Microarray-based analysis of stress-regulated microRNAs in *Arabidopsis thaliana*. RNA.

[34] Qin DD, Wu HY, Peng HR, Yao YY, Ni ZF, Li ZX, Zhou CL, Sun QX (2008). Heat stress-responsive transcriptome analysis in heat susceptible and tolerant wheat (*Triticum aestivum* L.) by using Wheat Genome Array. BMC Genom..

[35] Schwab R, Palatnik JF, Riester M, Schommer C, Schmid M, Weigel D (2005). Specific effects of MicroRNAs on the Plant Transcriptome. Dev. Cell.

[36] Liu J, Cheng XL, Liu P, Sun JQ (2017). miR156-targeted SBP-box transcription factors interact with DWARF53 to regulate TEOSINTE BRANCHED1 and BARREN STALK1 expression in bread wheat. Plant Physiol..

[37] Johnson M, Zaretskaya I, Raytselis Y, Merezhuk Y, McGinnis S, Madden TL (2008). NCBI BLAST: A better web interface. Nucleic Acids Res..

[38] Shan QW, Wang YP, Li J, Gao CX (2014). Genome editing in rice and wheat using the CRISPR/Cas system. Nat. Protoc. Erecipes Res..

[39] Wang YP, Tang HB, Debarry JD, Tan X, Li JP, Wang XY, Lee TH, Jin HZ, Marler B, Guo H, Kissinger JC, Paterson AH (2012). MCScanX: A toolkit for detection and evolutionary analysis of gene synteny and collinearity. Nucleic Acids Res..

[40] Zhu Y, Wu NN, Song WL, Yin GJ, Qin YJ, Yan YM, Hu YK (2014). Soybean (*Glycine max*) expansin gene superfamily origins: Segmental and tandem duplication events followed by divergent selection among subfamilies. BMC Plant Biol..

[41] Krzywinski M, Schein JI, Connors J, Gascoyne R, Horsman D, Jones SJ, Marra MA (2009). Circos: An information aesthetic for comparative genomics. Genome Res..

[42] Thompson, J.D., Gibson, T.J. & Higgins, D.G. Multiple sequence alignment using ClustalW and ClustalX. *Curr. Protoc. Bioinform*. Chapter 2, Unit 2.3.1–2.3.22 (2002). 10.1002/0471250953.bi0203s00 18792934

[43] Kumar S, Stecher G, Tamura K (2016). MEGA7: Molecular evolutionary genetics analysis version 7.0 for bigger datasets. Mol. Biol. Evol..

[44] Hu B, Jin JP, Guo AY, Zhang H, Luo JC, Gao G (2015). GSDS 2.0: An upgraded gene feature visualization server. Bioinformatics.

[45] Bailey TL, Johnson J, Grant CE, Noble WS (2015). The MEME suite. Nucleic Acids Res..

[46] Pingault L, Choulet F, Alberti A, Glover N, Wincker P, Feuillet C, Eversole P (2015). Deep transcriptome sequencing provides new insights into the structural and functional organization of the wheat genome. Genome Biol..

[47] Li Z, Liu D, Xia Y, Li ZL, Jing DD, Du JJ, Niu N, Ma SC, Wang JW, Song YL, Yang ZQ, Zhang GS (2020). Identification of the WUSCHEL-related homeobox (WOX) gene family, and interaction and functional analysis of TaWOX9 and TaWUS in wheat. Int. J. Mol. Sci..

[48] Ramesh SA, Kamran M, Sullivan W, Chirkova L, Okamoto M, Degryse F, McLaughlin M, Gilliham M, Tyerman SD (2018). Aluminum-activated malate transporters can facilitate GABA transport. Plant Cell..

[49] Livak KJ, Schmittgen TD (2001). Analysis of relative gene expression data using real-time quantitative PCR and the 2 ^−ΔΔ C T^ method. Methods.

