# Supplementary material for: Genome-wide identification, characterization, and expression patterns analysis of the SBP-box gene family in wheat (Triticum aestivum L.)
Source: Sci Rep. 2020 Oct 14;10:17250. doi: 10.1038/s41598-020-74417-x (PMC7560695; doi:10.1038/s41598-020-74417-x)
Supplement: Supplementary file 1 — Supplementary Information. [file 41598_2020_74417_MOESM1_ESM.pdf]

Genome-wide identification, characterization, and expression patterns analysis of the SBP-box gene family in wheat (*Triticum aestivum* L.)

Ying Li[1,2,3,4,5], Qilu Song[1,2,3,4,5], Yamin Zhang[1,2,3,4,5], Zheng Li[1,2,3,4,5], Jialin Guo[1,2,3,4,5], Xinhong Chen[1,2,3,4,5]* and Gaisheng Zhang[1,2,3,4,5]*

[1]College of Agronomy, Northwest A & F University, Yangling, Shaanxi, 712100, P.R. China. [2]National Yangling Agricultural Biotechnology & Breeding Center, Yangling, Shaanxi, 712100, P.R. China. [3]Yangling Branch of State Wheat Improvement Centre, Yangling, Shaanxi, 712100, P.R. China. [4]Wheat Breeding Engineering Research Center, Ministry of Education, Yangling, Shaanxi, 712100, P.R. China. [5]Key Laboratory of Crop Heterosis of Shaanxi Province, Yangling, Shaanxi, 712100, P.R. China.

*** Correspondence:** cxh2089@126.com, zhanggaisheng18@sohu.com

Table S1: The detailed information of TaSBPs

Gene Name	Gene ID	Chrom no	Location		Properties of proteins		pI	GRAVY	Subcell location	EST valadation
					Protein length (aa)	Molecular weight (Da)				
TraesCS1A02G255300	TaSBP1A	1A	447623204	447635025	859	94154.99	5.84	-0.299	Nuclear	34
TraesCS1B02G266100	TaSBP1B	1B	467540751	467553928	861	94260.18	5.83	-0.293	Nuclear	34
TraesCS1D02G254700	TaSBP1D	1D	347199194	347210946	859	93957.68	5.81	-0.308	Nuclear	34
TraesCS2A02G232400	TaSBP2A	2A	276276336	276279155	192	20127.47	9.87	-0.611	Nuclear	28
TraesCS2B02G250900	TaSBP2B	2B	260765427	260768180	192	20117.43	9.87	-0.607	Nuclear	31
TraesCS2D02G232800	TaSBP2D	2D	206632153	206636152	192	20129.44	9.87	-0.625	Nuclear	28
TraesCS2A02G413900	TaSBP3A	2A	670957294	670962629	349	36409.02	9.47	-0.383	Nuclear	13
TraesCS2D02G410700	TaSBP3D	2D	525512403	525517689	355	36947.6	9.39	-0.385	Nuclear	12
TraesCS2B02G530400	TaSBP4B	2B	725575939	725580611	408	43960.41	7.24	-0.758	Nuclear	1
TraesCS2A02G502300	TaSBP4A	2A	730613571	730617739	412	44489	7.26	-0.779	Nuclear	0
TraesCS2D02G502900	TaSBP4D	2D	596550011	596554762	407	43872.39	7.79	-0.751	Nuclear	1
TraesCS3A02G432500	TaSBP5A	3A	673898993	673902601	415	43741.55	8.93	-0.548	Nuclear	14
TraesCS3B02G468400	TaSBP5B	3B	713320663	713324953	414	43481.25	8.93	-0.518	Nuclear	16
TraesCS3D02G425800	TaSBP5D	3D	538401064	538404375	419	44183.04	8.93	-0.529	Nuclear	18
TraesCS4A02G359500	TaSBP6A	4A	632857804	632864321	962	105372.91	5.73	-0.306	Nuclear	52
TraesCS5A02G286700	TaSBP7A	5A	494569538	494575755	430	44923.11	6.88	-0.395	Nuclear	13
TraesCS5B02G286000	TaSBP7B	5B	471401006	471406978	433	45255.54	6.88	-0.381	Nuclear	13
TraesCS5D02G294400	TaSBP7D	5D	391372552	391378851	432	45253.53	6.88	-0.373	Nuclear	14
TraesCS5B02G265600	TaSBP8B	5B	450105102	450108305	487	45151.6	8.87	-0.66	Nuclear	3
TraesCS6A02G110100	TaSBP9A	6A	79267605	79271671	475	51411.39	9.18	-0.651	Nuclear	20
TraesCS6B02G138400	TaSBP9B	6B	135858620	135862931	381	41620.78	9.7	-0.713	Nuclear	30
TraesCS6D02G098500	TaSBP9D	6D	62113576	62118381	473	51243.17	9.19	-0.662	Nuclear	30
TraesCS6A02G155300	TaSBP10A	6A	143965449	143969690	328	35420.76	9.34	-0.629	Nuclear	9
TraesCS6B02G183400	TaSBP10B	6B	204923634	204927878	327	35317.66	9.34	-0.664	Nuclear	9
TraesCS6D02G145200	TaSBP10D	6D	115545817	115550011	325	35190.52	9.25	-0.639	Nuclear	20
TraesCS6A02G152000	TaSBP11A	6A	136541404	136544531	448	48092.44	6.54	-0.608	Nuclear	0
TraesCS6B02G180300	TaSBP11B	6B	200508894	200512052	442	47838.07	7.02	-0.682	Nuclear	0
TraesCS6D02G142100	TaSBP11D	6D	111567310	111570133	452	48862.29	7.27	-0.659	Nuclear	0
TraesCS7A02G494800	TaSBP12A	7A	684292548	684297219	419	45396.55	8.98	-0.399	Nuclear	2
TraesCS7D02G482200	TaSBP12D	7D	592632237	592634499	408	44132.98	9.86	-0.547	Nuclear	2
TraesCS7A02G495000	TaSBP13A	7A	685212558	685214906	406	43554.82	9.23	-0.57	Nuclear	6
TraesCS7B02G402300	TaSBP13B	7B	668928550	668930740	390	42165.25	9.3	-0.627	Nuclear	2
TraesCS7D02G482400	TaSBP13D	7D	592816284	592819509	388	41770.7	8.78	-0.589	Nuclear	3
TraesCS7A02G494900	TaSBP14A	7A	685090082	685095954	394	42655.9	9.16	-0.578	Nuclear	6
TraesCS7B02G402400	TaSBP14B	7B	669139219	669141554	401	43537.92	9.44	-0.624	Nuclear	4
TraesCS7D02G482300	TaSBP14D	7D	592677316	592679805	405	43409.77	9.17	-0.566	Nuclear	2
TraesCS7A02G260500	TaSBP15A	7A	252715392	252720978	407	43226.94	6.51	-0.532	Nuclear	14
TraesCS7B02G158500	TaSBP15B	7B	214070642	214075881	409	43463.29	6.51	-0.502	Nuclear	14
TraesCS7D02G261500	TaSBP15D	7D	237410146	237416092	414	43725.52	7.08	-0.495	Nuclear	14
TraesCS7B02G142200	TaSBP16B	7B	181033715	181039167	1104	120626.35	7.19	-0.52	Nuclear	85
TraesCS7D02G248000	TaSBP16D	7D	219291031	219296565	1124	123141.26	7.38	-0.528	Nuclear	86
TraesCS7B02G144900	TaSBP17B	7B	187777243	187781491	386	40242.21	8.97	-0.751	Nuclear	5
TraesCS7D02G245200	TaSBP17D	7D	213786904	213791354	385	40205.21	9.13	-0.759	Nuclear	5
TraesCS7A02G246500	TaSBP17A	7A	225631628	225636261	386	40281.35	9.02	-0.751	Nuclear	5
TraesCS7B02G115200	TaSBP18B	7B	133742358	133748024	845	93295.12	8.45	-0.314	Nuclear	13
TraesCS7D02G210400	TASBP18D	7D	168423642	168428099	847	93664.32	8.64	-0.344	Nuclear	13
TraesCS7A02G495100	TaSBP19A	7A	685227875	685230770	418	44452.59	8.95	-0.627	Nuclear	9
TraesCS7B02G402200	TaSBP19D	7B	668907244	668914987	416	44134.14	9.05	-0.647	Nuclear	8

Table S2 Wheat SBP gene duplication events

Duplication type	Gene ID	Chrom no	Location		Gene ID	Chrom no	Location	
tandem duplication	TaSBP12D	7D	592632237	592634499	TaSBP14D	7D	592677316	592679805
	TaSBP13B	7B	668928550	668930740	TaSBP14B	7B	669139219	669141554
	TaSBP14A	7A	685090082	685095954	TaSBP13A	7A	685212558	685214906
segmental duplication	TaSBP1A	1A	447623204	447635025	TaSBP1B	1B	467540751	467553928
	TaSBP1A	1A	447623204	447635025	TaSBP1D	1D	347199194	347210946
	TaSBP1D	1D	347199194	347210946	TaSBP2A	2A	276276336	276279155
	TaSBP1D	1D	347199194	347210946	TaSBP2B	2B	260765427	260768180
	TaSBP2A	2A	276276336	276279155	TaSBP2D	2D	206632153	206636152
	TaSBP3A	2A	670957294	670962629	TaSBP3D	2D	525512403	525517689
	TaSBP4B	2B	725575939	725580611	TaSBP4D	2D	596550011	596554762
	TaSBP4B	2B	725575939	725580611	TaSBP4A	2A	730613571	730617739
	TaSBP5A	3A	673898993	673902601	TaSBP5B	3B	713320663	713324953
	TaSBP5A	3A	673898993	673902601	TaSBP5D	3D	538401064	538404375
	TaSBP7A	5A	494569538	494575755	TaSBP15A	7A	252715392	252720978
	TaSBP7D	5D	391372552	391378851	TaSBP15A	7A	252715392	252720978
	TaSBP7D	5D	391372552	391378851	TaSBP15D	7D	237410146	237416092
	TaSBP11B	6B	200508894	200512052	TaSBP19A	7A	685227875	685230770
	TaSBP11D	6D	111567310	111570133	TaSBP19A	7A	685227875	685230770
	TaSBP11D	6D	111567310	111570133	TaSBP14B	7B	669139219	669141554

Table S3: The primers used in this study

Gene ID	Forward	Reverse
TaSBP1A/B/D	AGCTTGCAAAATGACCGAAG	CATTTTCCTGTTTTGCACCA
TaSBP4B/D	ACCAAAGGCAAAGCAGCCGGAA	TCTTTCACCACCTCGCCGAGT
TaSBP6A	TCAGGCTAAGATGTACCCCAA	GTATCCCAACACAAACGCAGA
TaSBP9A/D	GGCTCCTTATGTTCAAATGA	TGTGTGTAACTTTGAAGCCTC
TaSBP9B	AGTTCAGATTTGCGCCTGTCA	GTTCCAGTCCTCGTGTTGTCA
TaSBP10A	GCTGTCCGATCACAATGCT	TGCCGTCTATCATCAAACACC
TaSBP16A	ATGCGCTTCTGCCAACAGT	TGCCAGTTTTGCTCCGGTCT
TaSBP17B/D	CCAGCGCCAAGGATTACCAC	TGCATCTGGTTGCCAACTACCG
TaSBP18A/B/D	CAGCTCGTCCAACTGGTCT	GGGCTTCTTCAGTTCCATGCTT
TaSBP19B/D	ATCATCCAAGGCTCCGCAAC	TGTCCAGCAGCATCTTTACCAC
Taactin	CACACTGGTGTTATGGTAGG	AGAAGGTGTGATGCCAAAT
